# Acute Phrenic Neuropathy and Diaphragmatic Dysfunction as a Complication of COVID-19: A Report of Four Cases

**DOI:** 10.7759/cureus.34419

**Published:** 2023-01-30

**Authors:** Juan J Medina-Pérez, José A Balderas-Juárez, Andrés Vega-Rosas, Paola G Ballesteros-Penedo, Silvia G Coubert-Pelayo

**Affiliations:** 1 Pain Management, Angeles Mocel Hospital, Mexico City, MEX; 2 Neurology, IMSS (Instituto Mexicano del Seguro Social) Venados Hospital, Mexico City, MEX; 3 Mental Health, Angeles Mocel Hospital, Mexico City, MEX; 4 Pulmonology, National Institute of Respiratory Diseases, Mexico City, MEX

**Keywords:** neuropathy, diaphragm, phrenic nerve, sars-cov-2, covid-19

## Abstract

Among the neurological manifestations associated with coronavirus disease 2019 (COVID-19), neuropathies are rare. They have been associated with prolonged prostration and metabolic failure in a seriously ill patient. We present a case series of four Mexican patients diagnosed with diaphragmatic dysfunction due to phrenic neuropathy during acute COVID-19, documented by conduction velocities of the phrenic nerves. Blood tests, chest computed tomography (CT), and nerve conduction velocities of the phrenic nerves were performed. COVID-19 patients with phrenic nerve neuropathy represent a therapeutic challenge since they have high oxygen requirements due to the malfunction of ventilatory mechanics secondary to neuromuscular damage, as well as the damage that pneumonia generates in lung tissue. We confirm and extend the neurological manifestations of COVID-19, the impact on the neuromuscular dysfunction of the diaphragm, and its consequences such as the difficulty of weaning from mechanical ventilation.

## Introduction

Neuropathy has been described as one of the neurological manifestations of the severe acute respiratory syndrome coronavirus 2 (SARS-CoV-2) infection in coronavirus disease 2019 (COVID-19). SARS-CoV-2 infection can cause several neurological manifestations, including neuropathies [1]; however, it represents an important problem due to its acute consequences that can significantly increase mortality. Inflammatory olfactory neuropathy [2], motor peripheral neuropathy [3] and neuromuscular involvement in COVID-19 critically ill patients [4] are some examples of the multiple reports from around the world of the ability of SARS-CoV-2 to generate damage to the peripheral nervous system. On the other hand, the main causes of hypoxemia in patients diagnosed with COVID-19 are due to the damage caused by the viral infection in the lung parenchyma. In the present series of cases, we expose the neuropathic damage that SARS-CoV-2 can generate on the neural circuits responsible for the innervation of the main respiratory muscle, the diaphragm. 

Currently, there are several reports of neuromuscular disease due to COVID-19 [5]; however, documented cases of diaphragm neuropathy are very rare, causing rapid deterioration of respiratory mechanics in COVID-19 patients [6]. Borroni et al. reported two COVID-19 patients with focal diaphragmatic myoclonus (DM) [7]. With the present observation, we report four cases of diaphragmatic dysfunction due to phrenic nerve (PN) neuropathy, as a neurological acute complication of SARS-CoV-2 infection.

The cases of four patients are included in this report. The patients were adults with an average age of 60.5 ± 8.5 (range: 52-69 years), with COVID-19 documented diagnosis, who had a severe clinical presentation upon hospital admission. All the patients sought consultations between December 2020 and March 2021 in two different hospitals in Mexico City, where the dysfunction of their PNs was studied after signing informed consent for the procedures performed. During their hospitalization, each patient underwent the following evaluations: nasopharyngeal swab testing for SARS-CoV-2 with real-time polymerase chain reaction assay (RT-PCR) to confirm the disease, D-Dimer, ferritin, creatine phosphokinase (CPK), creatine phosphokinase-MB (CPK-MB), Clauss fibrinogen assay (CFA), general blood tests, chest CT, and bilateral nerve conduction velocities (NCV) of the PNs to document neuronal damage. None of the patients had any vaccination against SARS-CoV-2 at the time of contracting the infection.

## Case presentation

Case 1

A 56-year-old male patient with a history of poorly controlled type 2 diabetes mellitus (T2DM), systemic arterial hypertension (SAH) under treatment, and obesity grade 1; suffered headache, anosmia, ageusia, non-productive cough, myalgia, and non-quantified fever as initial symptoms. After eight days of increased severity, he presented to the Emergency Room (ER) with oxygen saturation (SpO2) of 89% in room air, blood pressure of 176/104 mmHg, tachycardia (114 bpm), tachypnea (26 rr), and a temperature of 37°C. Hospitalization was decided and the diagnosis of SARS-CoV-2 infection was made by RT-PCR. Anticoagulant, steroid anti-inflammatory, insulin, and antihypertensive treatment was started. During hospitalization, he presented extreme sinus bradycardia and increased oxygen requirement with thoracoabdominal dissociation dependent on the diaphragmatic musculature. NCV was performed, finding bilateral blocked nerve conduction of PN with right predominance, as well as decreased amplitude and voltage and prolonged latencies. Subsequently, tenofovir/emtricitabine 245/200 mg every 24 hours, ruxolitinib 15 mg every 12 hours, and exercise-based pulmonary rehabilitation (incentive spirometry and hammer vest) were added, normalizing diaphragmatic breathing patterns and achieving his discharge after 28 days of hospital stay.

Case 2

A 55-year-old female patient with a history of poorly controlled TD2M and SAH under treatment; began suffering from productive cough, asthenia, and adynamia as initial symptoms. Eight days later, she presented to the ER with a fever of 39°C and SpO2 of 69% in room air, blood pressure of 136/83 mmHg, tachycardia (135 bpm), and tachypnea (25 rr). Hospitalization was decided and the diagnosis of SARS-CoV-2 infection was made by RT-PCR. She was given anticoagulants, anti-inflammatory steroids, insulin, and antihypertensive and ascorbic acid treatment. A torpid evolution occurred in the form of an increase in respiratory effort and desaturation, for which non-invasive ventilation (NIV) was given with partial improvement. Notwithstanding positive airway pressure support, advanced management of the airway was decided. Neurology management was requested due to polyneuropathy in the critical state and physical rehabilitation was initiated. General NCV were performed including PNs, finding mixed polyneuropathy that symmetrically affected the four extremities, predominantly the lower ones, and there was an absence of response from both PNs (absence of bilateral compound muscle action potential). Invasive ventilation (IV) had poor pulmonary response, low compliance, and difficulty reaching oxygenation targets, even in pronation. She developed septic shock due to bacterial pneumonia secondary to health care, deterioration to multiple organ failure, high vasopressor requirements, repeated events of bronchospasm with severe hemodynamic repercussions, bradycardia, and loss of sphincters control; she passed away on day 49 of hospital stay.

Case 3

A 69-year-old female patient with a history of poorly-controlled T2DM, SAH, and hypothyroidism, both under treatment, began suffering from diarrhea, fever, and prostration, for which a physician initiated quinolone-based management, under suspicion of urinary tract infection and a negative SARS-CoV-2 antigen test. Seventy-two hours later, she presented intolerance to the oral route and SpO2 of 49%, for which she was transferred to the ER with a blood pressure of 125/75 mmHg, tachycardia (105 bpm), tachypnea (28 rr), and temperature of 36.2°C. Hospitalization was decided and COVID-19 diagnosis was made by RT-PCR. Anticoagulants, steroids, levothyroxine, insulin, antihypertensive, and antibiotics were started. She showed rapid neurological deterioration, for which a brain MRI was performed, finding loss of parenchymal volume predominantly in the frontal and parietal lobes, compensatory ventriculomegaly, gliosis associated with multi-infarct vascular disease, and a significant decrease in the caliber and intensity of the mean brain arteries signal. Additionally, hypertensive heart disease, degenerative mitral regurgitation, and a high probability of pulmonary hypertension were reported by echocardiography. On the third day of the hospital stay, she presented an increase in respiratory effort and desaturation, for which NIV was given with high-dose corticosteroids and O_2_ by high-flow nasal cannula (HFNC) and early switched to IV for 24 days. On the 19th day, electromyography was performed, finding evidence of moderate to severe sensory-motor peripheral polyneuropathy of a mixed, axonal, and demyelinating type that affected all four extremities, with a predominance of the lower ones, for which physical therapy and rehabilitation was initiated based on active-assisted mobilizations and electrostimulation. One week later, presented motor dysphagia and NCV of PN were performed, reported as abnormal, due to the presence of a moderate prolongation of terminal latencies and a relative decrease in the amplitude of bilateral responses, predominately on the left nerve, with evidence of segmental and trunk demyelination, for which swallowing rehabilitation was practiced for 24 days and application of supramaximal electrostimulation in diaphragmatic muscles (SEM) for 10 days and incentive spirometry until after hospital discharge. The patient presented a better evolution and was discharged due to improvement after 58 days of hospital stay. 

Case 4

A 52-year-old male, with no significant clinical history, began suffering from general malaise, asthenia, adynamia, headache, hyporexia, and mild respiratory symptoms. SARS-CoV-2 RT-PCR was performed with positive results. One week later, he progressed to respiratory failure and desaturation, attending an ER assessment where COVID-19 Reporting and Data System (CO-RADS) 6 atypical pneumonia was diagnosed, with a 13/25 severity score, requiring hospitalization. After two days, he presented confusional syndrome as well as increased work of breathing and use of accessory muscles of respiration, SpO2 of 74%, requiring O2 with HFNC with 60 L/min and fraction of inspired oxygen (FiO2) 100%, maintaining 95% arterial oxygen saturation (SaO2), oxygen pressure targets (pO2>60 mmHg), and no tomographic progression of pneumonia. Despite this, the patient remained polypneic (34 rr), being admitted to the intensive care unit (ICU) for surveillance. He evolved hours later with tachypnea up to 38 bpm and SpO2 88% with HFN. In the neurological evaluation, paroxysmal (diaphragmatic) abdominal myoclonus was documented, which affects ventilatory mechanics (VM), therefore, IV support was given. Neurophysiological studies were requested among the normal electroencephalogram (EEG) and phrenic NCVs, reporting severe axonal bilaterally neuropathy, considering the possibility of a postinfectious peripheral neurological complication of COVID-19. Physical rehabilitation therapy was started with electrostimulation of the PN with subcutaneous electrodes of both hemidiaphragms, negative pressure chest shield was placed for pulmonary, levetiracetam was started for myoclonus, and diaphragmatic rehabilitation (including SEM) and a tracheostomy. He was treated with dexamethasone, ceftriaxone, linezolid, vancomycin, enoxaparin, ipratropium bromide, budesonide, acetylcysteine, and clonazepam throughout the illness. The patient was kept under multidisciplinary treatment for two months, improving his neurological condition as well as the stability of the VM without abdominal myoclonus. Subsequently, NCV was repeated, reporting improvement in the latencies and amplitudes of both PNs and finally, the patient could be extubated and progressed from IV. The patient presented a better evolution and was discharged due to improvement after 67 days of hospital stay.

The main laboratory test results of the first 24 hours of the hospital admission of the four patients treated in two different hospital centers are given in Table 1.

**Table 1:** Laboratory studies in the first 24 hours of hospital stay CPK: creatine phosphokinase; CPK-MB: creatine phosphokinase-MB; CFA: Clauss fibrinogen assay Note: Due to logistical reasons, it was not possible to obtain all the values ​​from the clinical record of patient 4.

Variable	Patient 1	Patient 2	Patient 3	Patient 4	Reference range	Unit
Glucose	284	301	323	110	70-99	mg/dL
D-Dimer	585	1144	3365	847	<500	ng/mL
Ferritin	627.4	443.8	4086.2	-	12-300	ng/mL
CPK	36	27.53	271.49	-	0-5	ng/mL
CPK-MB	0.2	0.7	2.7	-	0-5	ng/mL
CFA	843	1016	553	-	200-400	mg/dL

## Discussion

The most common clinical presentation of severe COVID-19 is acute respiratory failure consistent with acute respiratory distress syndrome (ARDS) [8]. Many patients hospitalized with severe pneumonia due to COVID-19 show hypoxemia without clinical signs of dyspnea, a condition which has been coined ‘happy hypoxemia’. In a limited number of patients, a sudden deterioration occurs during the course of illness, with an increase in oxygen demand when fatigue and dyspnea set in. We hypothesized that dysfunction of the diaphragm could be a contributing factor in the clinical course of patients requiring admission to the ICU [9].

**Figure 1:**
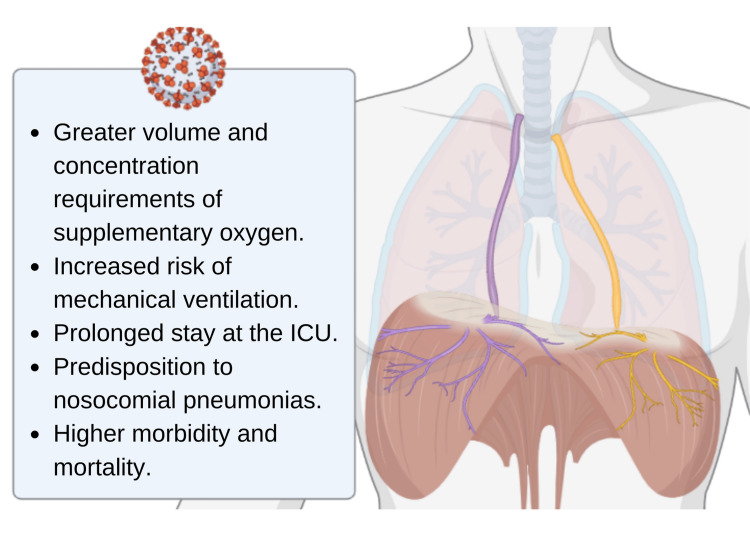
Problems associated with acute neuropathy of phrenic nerves due to COVID-19 COVID-19: coronavirus disease 2019l ICU: intensive care unit Image Credit: Authors, with BioRender software

In this report, we described the cases of four patients with COVID-19 diagnosis who presented with acute diaphragmatic neuromuscular dysfunction secondary to phrenic neuropathy. COVID-19 patients with PN damage represent a therapeutic challenge since they have high oxygen requirements due to the affection of VM secondary to neuromuscular damage, in addition to the damage of the lung parenchyma that causes viral pneumonia. MV dysfunction causes higher supplemental oxygen requirements, associated with a greater demand for volume and concentration to maintain SpO2; increasing the risk of IV, prolonged stay in the intensive respiratory care unit, predisposing to other nosocomial pneumonias and higher morbidity and mortality (Figure 1). On the other hand, the neurotropic capacity of SARS-CoV-2 is well known, as well as its ability to infect the nervous system and generate different central and peripheral manifestations, such as neuropathies [3-5].

The four cases had very peculiar characteristics. Cases 2-4 presented bilateral dysfunction of the PNs, which improved with combined rehabilitation therapy (electrostimulation, external negative pressure, incentive spirometer, etc.). Farr et al. reported a cohort of 21 COVID-19 patients who manifested diaphragmatic dysfunction after IV, and in the post-extubation period manifested post-ventilator diaphragm dysfunction, post-intensive care syndrome, and critical illness myopathy; as well as muscle membrane dysfunction, microcirculatory changes associated with inflammation, and impaired glucose transporter type 4 translocation to the muscle membranes [10]. Borroni et al.reported two cases where DM was not accompanied by evidence of structural damage of the central nervous system through EEG use, where abnormalities were reported without determining that it was cortical myoclonus [7]. In Case 4 of the current report, no abnormal activity was documented in the EEG study, and cortical origin was ruled out, which, as reported in the literature, cannot rule out a causal association with COVID-19.

It is worth mentioning that we only describe four patients; therefore, it is difficult to make a generalization. This opens the doors to a more in-depth prospective study with a larger number of patients. Another limitation of our study is the absence of diaphragmatic ultrasound studies that help us determine parameters such as diaphragmatic excursion time and percentage of diaphragmatic thickening, which in turn would have allowed us to determine treatment decisions (intubation or early tracheostomy) as well as functional prognosis of our patients. Finally, the information that arises from neurological complications due to COVID-19 is extensive and in the future, we will be able to consolidate the information necessary for decision-making.

## Conclusions

In the cases in this report, the temporal sequence suggests but does not prove, that SARS-CoV-2 was a causal factor of PN neuropathy and DM, enriching the knowledge we have about the neurological manifestations of COVID-19. This case series improves the understanding of the sequelae of diaphragmatic paralysis and analyzes whether they are associated with acute infection, since the increased oxygen requirement occurred around eight days after hospital admission. Vaccination has improved morbidity and mortality by decreasing the viral load in patients who have contracted the infection, so considering this type of neurological manifestations in T2DM patients, with an incomplete vaccination schedule or with more than nine months since the last booster, are factors to consider. Finally, promoting multidisciplinary work in the approach to these types of cases and incorporating therapeutic interventions with respiratory physiotherapy and targeted rehabilitation, can offer patients a better prognosis.
